# The histological and biochemical analysis of the effects of radiofrequency radiation on testis tissue of rats and the protective effect of melatonin

**DOI:** 10.55730/1300-0144.5857

**Published:** 2024-03-04

**Authors:** Armağan YARDIM, Bahriye SIRAV, Arın TOMRUK, Sinem ORUÇ, Kevser DELEN, Dilek KUZAY, Cemile Merve SEYMEN, Gülnur TAKE KAPLANOĞLU

**Affiliations:** 1 Department of Biophysics, Faculty of Medicine, Gazi University, Ankara, Turkiye; 2 Department of Biophysics, Faculty of Medicine, Fırat University, Elazığ, Turkiye; 3 Department of Neurology, Gülhane Training and Research Hospital, University of Health Science, Ankara, Turkiye; 4 Department of Physiology, Faculty of Medicine, Ahi Evran University, Kırşehir, Turkiye; 5 Department of Histology and Embryology, Faculty of Medicine, Gazi University, Ankara, Turkiye

**Keywords:** Radiofrequency, reactive oxygen species, hematoxylin-eosin, testis, rat

## Abstract

**Background/aim:**

Primarily due to wireless communication devices, especially mobile phones, there has been a steady rise in the intensity of nonionizing radiofrequency radiation (RFR). In recent years, increased human health problems raised concerns about whether there is a positive relationship between intense exposure to RFR and public health. The present study aims to investigate the effects of GSM-like RFR exposure on the male reproductive system and the impact of melatonin treatment (synergistic, antagonist, or additive).

**Materials and methods:**

Thirty-six male Wistar Albino rats were used and separated into six groups: i. Control; ii. Sham; iii. RFR exposure; iv. Control-melatonin; v. Sham-melatonin; vi. Melatonin + RFR exposure. Animals were exposed to 2600 MHz RFR with electric (E) field levels of 21.74 V/m for 30 min per day, 5 days per week, for 4 weeks. All testicular tissue samples were evaluated under a light microscope for hematoxylin-eosin staining. Biochemical analyses were performed by measuring malondialdehyde, total nitric oxide, glutathione, and glutathione peroxidase levels. We evaluated the combined effects of prolonged RFR exposure and melatonin treatment on ROS-mediated structural changes in testicular tissues.

**Results:**

Results showed that reactive intermediates (malondialdehyde and total nitric oxide) increased significantly with RFR exposure, while the protective effect of melatonin effectively reduced the radical levels of the tissues. Histological evaluation revealed a decrease in cell population and connective tissue elements under RFR exposure, accompanied by marked edema in the testicular tissues.

**Conclusion:**

The structural and functional effects of prolonged RFR exposure might be ROS-based. Moreover, these adverse effects might be compensated with externally treated supplements. There is a need for new extensive research.

## 1. Introduction

There has been a steady increase in the intensity of radiofrequency radiation (RFR) in the environment due to mobile communication devices. Growing health issues among humans have raised concerns regarding the potential correlation between exposure to RFR and human health. The biological effects of RFR depend on some parameters, such as frequency, polarization, intensity, exposure duration, exposed objects, and dielectric properties of exposed material. The interactions of RFR may lead to structural changes in biomolecules and alterations in the regulatory cascades of biochemical processes [1].

RFR (30 kHz to 300 GHz) is involved in the nonionizing part of the electromagnetic (EM) spectrum. Because of its insufficient energy for direct ionization of molecules, structural changes in molecules can be observed via the secondary messengers or reactive oxygen/nitrogen intermediates produced by nonionizing RFR [2]. With the depolarization of the mitochondrial membrane induced by RF exposure, the free reactive oxygen and hydroxyl radicals are formed as the primary sources of degenerative intermediates [3].

Various RF-induced effects on the male reproductive system, primarily focusing on testicular tissue cells, are available in the literature [4–10]. The fundamental mechanism of how RFR affects the reproductive organs in humans has yet to be fully elucidated. Most studies argue that overproduced cellular free radicals after RFR exposure may increase the sensitivity of male reproductive tissues and decrease the quality of sperm capacity by catalyzing redox reactions [11].

The essential impact of exogenous melatonin treatment is expected to be on the electron transport chain as the antioxidant properties. The detoxification of overproduced free radicals by increasing the activities of the antioxidant defense system can have an essential effect on the human body and reproductive systems [12–16]. Melatonin produced by the central nervous and reproductive systems has different impacts on the anterior pituitary gonadotropins, gonadal steroids, and testicular functions [17].

The present study aims to investigate the effects of 2600 MHz GSM-like RFR exposure in the male reproductive system and the role of melatonin treatment (synergistic, antagonist, or additive). The 2600 MHz RFR frequency was used to investigate the frequency of the 5th generation of mobile systems, and there are limited studies available concerning this specific frequency. GSM-like RFR exposure simulates the RFR exposures of mobile phones. Free radical-mediated structural changes in the primary constituent of the male reproductive and antioxidant defense system were analyzed under combined treatments of melatonin and RFR.

## 2. Materials and methods

Ethical approval was taken for 6 animals per group from Gazi University Local Ethics Committee for Animal Experiments (G.U.ET-22.046). This number of experimental animals is allowed for experiments by the Ethics Committee.

Adult male Wistar Albino rats (12–16 weeks old, weighing 250 ± 30 g) were obtained from the Laboratory Animals Raising and Experimental Research Center (GUDAM) in this study. Animals were housed under standard conditions (12 h light/12 h dark cycle with 22 ± 2 °C ambient temperature). Tap water and a regular diet were given to animals.

Thirty-six male Wistar Albino rats were separated into six groups (n = 6):

**Group I (control):** Animals maintained regular activity in their cage without application.
**Group II (sham):** Animals were placed in the exposure setup for 30 min per day, 5 days a week, over a period of 4 weeks, with no melatonin or RFR exposure.
**Group III (RFR):** Animals were exposed to RFR for 30 min per day, 5 days a week, over a period of 4 weeks.
**Group IV (melatonin):** Animals were subcutaneously injected with melatonin at a dose of 10 mg/kg/day for a month and housed in their usual living environment throughout the entire experiment.
**Group V (sham-melatonin):** Animals were subcutaneously injected with melatonin at a dose of 10 mg/kg per day for a month and then placed in the exposure setup for 30 min per day, 5days a week, over a period of 4 weeks, without RFR exposure.
**Group VI (melatonin+RFR)**: Animals were subcutaneously injected with melatonin at a dose of 10 mg/kg per day for a month and then exposed to RFR for 30 minutes per day, 5 days a week, over a period of 4 weeks.

**Figure 1:**
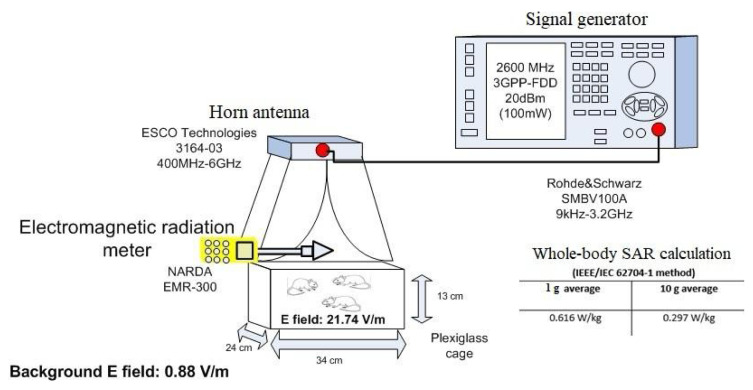
2600 MHz RFR exposure setup.

The RFR system is described in Figure 1 [6]. This system comprises a vector signal generator (Rohde &Schwarz, Munich, Germany) and a horn antenna (ETS Lingren, Cedar Park, TX). During exposure, rats were placed in plastic cages measuring 34 × 24 × 13 cm, with three animals per cage. The 2600 MHz RFR exposures were performed by positioning the horn antenna on top of the plastic cage. For RFR exposures, electric field levels were measured in the cage and found to be homogenous, so it was suitable to put 3 animals in the cage for each exposure. External and internal electromagnetic field strengths were measured using a Narda EMR 300 and its related probe during the exposure, and electric field levels were measured as follows:


$$\begin{array}{r} {\text{E}}_{\text{external}}:0.88\;\text{V}/\text{m }(\text{outside of the cage}),\\ {\text{E}}_{\text{internal}}:21.74\;\text{V}/\text{m }(\text{inside of the cage}). \end{array}$$

The whole-body SAR values were calculated as 0.616W/kg for a 1 g average and 0.297 W/kg for a 10 g average using the IEEE/IEC 62704-1 method [6].

After the last exposure, the rats were anesthetized with intramuscular injections of ketamine (35 mg/kg) and xylazine (5–10 mg/kg) and decapitated. Testis tissues were analyzed both histologically and biochemically.

### 2.1. Histological analysis

All testicular tissue samples were first fixed in 10% formaldehyde solution for light microscopic examination. After fixation, tissue samples were placed in cassettes and washed under running water for 24 h. To remove moisture, tissues were passed through increasing degrees of alcohol (70%, 80%, 90%, 100%). The tissues were then passed through xylol for polishing and embedded in molten paraffin. Hematoxylin-eosin staining was applied to the 4–5-micron thick sections obtained from the prepared paraffin blocks, and their pictures were taken by evaluating them in the LAS program.

### 2.2. Biochemical analysis

Malondialdehyde (MDA), total nitric oxide (NOx), glutathione (GSH), and glutathione peroxidase (GSH-Px) levels of the testicular tissues were studied by using a commercial ELISA kit (Shanghai Sunred Biological Technology, Shanghai, China). The absorbance of the samples was measured at 450 nm. Results were expressed as ng/mL for GSH-Px, nmol/g tissue for MDA, μmol/g tissue for NOx, and mol/g tissue for GSH.

### 2.3. Statistical analysis

SPSS 20.0 was used for the statistical analyses of the present study. The Kruskal–Wallis (nonparametric) test was applied to evaluate differences among all groups, while the Mann–Whitney test was used to evaluate differences between pairs of groups.

## 3. Results

### 3.1. Hematoxylin-eosin staining of testis tissues

**Figure 2:**
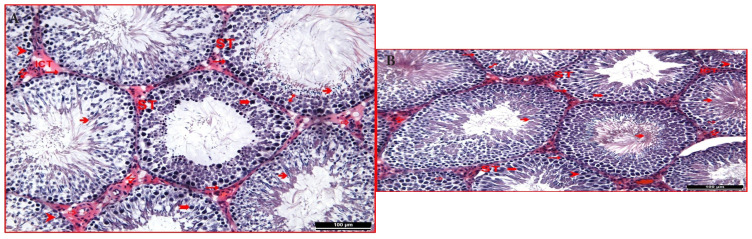
**(a)** In the testicular tissue of the control group, seminiferous tubule (ST), interstitial connective tissue (ICT), the germinal epithelium (1), spermatogonium (“), primary spermatocyte (°), spermium (â) Sertoli cell (†), capillaries (_), and Leydig cells (7) were observed (hematoxylin-eosin 200×). **(b)** In the testicular tissue of the sham group seminiferous tubule (ST), interstitial connective tissue (ICT), the germinal epithelium (1), spermatogonium (“), primary spermatocyte (°), spermium (â), Sertoli cell (†), capillaries (_), and Leydig cells(7) were observed (hematoxylin-eosin 200×).

Control and sham groups were observed with typical structures of the seminiferous tubules of the tissue and the interstitial connective tissue located between these tubules in hematoxylin-eosin staining of testicular tissue. Spermatogenic cells located in the germinal epithelium of the seminiferous tubule were notable. The primary spermatocytes, distinguished by their prominent appearance in the upper row, and the spermatogonia, with their tails oriented towards the lumen in the other layers, were particularly noticeable. It was observed that the lumen of the seminiferous tubule was densely filled with spermium. Again, among the spermatogenic cells, pyramidal-shaped Sertoli cells extending from basal to apical were observed with their typical structures. Numerous capillaries and interstitial (Leydig) cells in groups were seen in the interstitial space between the tubules with their usual forms (Figures 2a and 2b).

**Figure 3:**
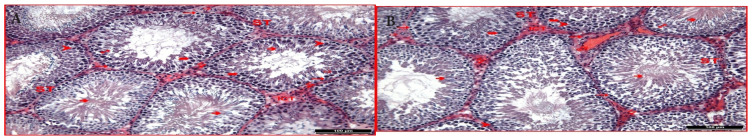
**(a)** In the control melatonin group testicular tissue, seminiferous tubule (ST), interstitial connective tissue (ICT), the germinal epithelium (1), spermatogonium (“), primary spermatocyte (°), spermium (â), Sertoli cell (†), capillaries (_), and Leydig cells (7) were observed (hematoxylin-eosin 200×). **(b)** In the sham melatonin group testicular tissue, seminiferous tubule (ST), interstitial connective tissue (ICT), the germinal epithelium (1), spermatogonium (“), primary spermatocyte (°), spermium (â), Sertoli cell (†), capillaries (_), and Leydig cells (7) are observed (hematoxylin-eosin 200×).

Control melatonin and sham melatonin groups were evaluated, and it was observed that the general structure was identical to the control and sham control groups. Unlike the control groups, it was noted that in the melatonin-administered groups, some seminiferous tubules contained relatively more spermatocytes, and there was a reasonably dense presence of spermium in the lumens of some tubules. This finding was interpreted to suggest that melatonin administration may have increased sperm production. In the sham control group examinations, it was also noted that some capillaries in the interstitial area were dilated (Figures 3a and 3b).

**Figure 4:**
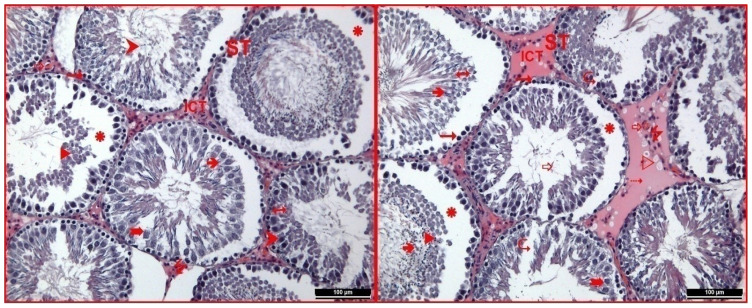
In the RFR group testicular tissue, seminiferous tubule (ST), interstitial connective tissue (ICT), germinal epithelium (1), spermatogonium (“), primary spermatocyte (°), spermium (â), Sertoli cell (†), capillaries (_), dilated capillaries (Y), Leydig cells (7), edema (ä), cells of the spermatogenic series emptied towards the lumen (u), regions where intercellular connections are broken (N), vacuolization (w) and hyalinization (4) were observed (hematoxylin-eosin 200×).

In the RFR group, it was noted that the seminiferous tubules did not exhibit any shape disorder, but there was intense edema in the germinal epithelium of the seminiferous tubule in most tubules. The standard impression and organization of the epithelium disappeared in the tubules with intense edema, although some spermatogonia retained their basal positions. Additionally, other cells of the spermatogenic series were found out of their regular arrangement and had emptied into the lumen. It was clearly distinguished that the intercellular connections were disrupted in the tubules. Again, it was noted that spermium in the lumens of some tubules was relatively reduced compared to other groups. Intensive vacuolization was observed in the examinations performed in the interstitial area of this group. In this area, it was observed that the cell population and connective tissue elements decreased relatively compared to other groups and gained a hyalinized appearance (Figure 4).

**Figure 5:**
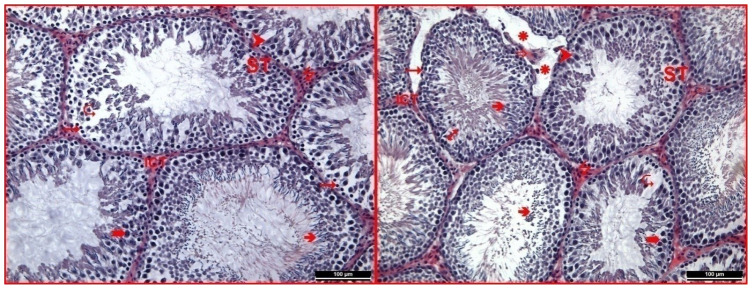
In RFR and melatonin group testicular tissue, seminiferous tubule (ST), interstitial connective tissue (ICT), germinal epithelium (1), spermatogonium (“), primary spermatocyte (°), spermium (â), Sertoli cell (†), capillaries (_), Leydig cells (7), edema (ä), and regions where intercellular connections are disrupted (N) were observed (hematoxylin-eosin 200×).

The hematoxylin-eosin staining of testicular tissue belonging to the RFR and melatonin group was described as the most striking finding. The general appearance exhibited a structure similar to the control and melatonin groups. On the other hand, it was observed that impaired connections between edema and spermatogenic series cells continued to be kept in some tubules. Again, edema was observed in the interstitial area in some regions. However, it was noted that the cell population in this area increased relatively compared to the RFR group (Figure 5).

### 3.2. Prooxidant and antioxidant levels of the tissues

**Figure 6:**
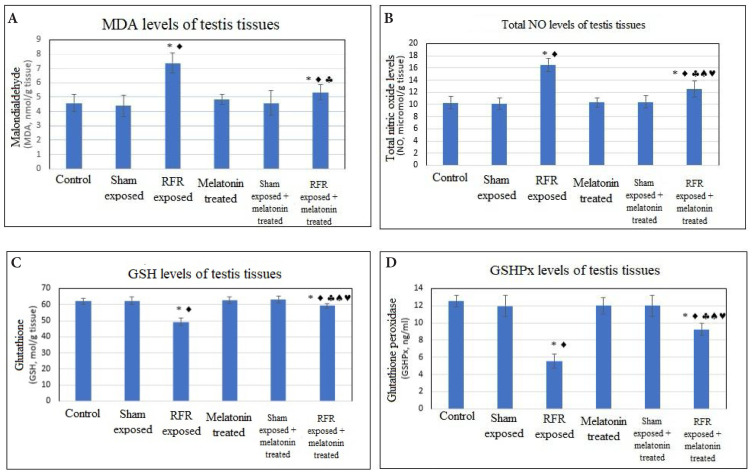
Prooxidant and antioxidant levels of testicular tissues under long-term exposure to 2600 MHz, for 30 min per day, 5 days per week, over a period of 4 weeks. All data are given as mean ± SD (p < 0.05) Intergroup comparisons. Groups vs. control: * Groups vs. sham: Groups vs. RFR exposed:§ Groups vs. melatonin treated:^a^ Groups vs. [sham exposed + melatonin treated]:©

The oxidative stress results obtained from all examined testicular tissues are given in Figure 6.

There was a significant increase in the level of lipid peroxidation with the effect of RFR exposure (7.37 ± 0.70) compared to the control (4.58 ± 0.58) and sham-exposed groups (4.40 ± 0.75) (p < 0.05).

External melatonin treatment significantly decreased the level of lipid peroxidation end product in the RFR + melatonin group (5.34 ±0.55) compared to the RFR group (7.37 ± 0.70) (p < 0.05).

Increased testicular nitric oxide levels were found in the RFR group (16.50 ± 1.10) compared to the control (10.30 ± 1.00) and sham-exposed groups (10.10 ± 0.98)(p < 0.05).

Melatonin treatment decreased the level of testicular nitric oxide for the RFR+melatonin group (12.53 ± 1.34) compared to the the RFR group (16.50 ± 1.10) (p < 0.05).

Testicular glutathione level decreased significantly in the RFR group (49.28 ± 2.36) compared to the control (62.00 ± 1.82) and sham-exposed groups (62.42 ± 2.47) (p < 0.05).

However, melatonin treatment increased the level of testicular glutathione for the RFR+melatonin group (59.22 ± 1.45) compared to the RFR group (49.28 ± 2.36) (p < 0.05).

Testicular antioxidant levels decreased significantly in the RFR group (5.5 ± 0.84) compared to the control (12.56 ± 0.65) and sham-exposed groups (11.97 ± 1.25) (p < 0.05).

Melatonin treatment increased the testicular glutathione level for the RFR+melatonin group (9.23±0.69) comared to the RFR group (5.5 ± 0.84) (p < 0.05).

## 4. Discussion

The present study investigated the possible histological and biochemical effects of RFR exposure in the male reproductive system and the role of melatonin. Results showed no structural disruption in the seminiferous tubules in male rats exposed to RFR. However, intense edema was observed in the germinal epithelium of the seminiferous tubule. The usual structural and functional properties of epithelium disappeared in the tubules with intense edema. It was observed that some spermatogonium retained their basal positions, but other cells that accumulated out of their regular structure moved into the lumen. Therefore, the intercellular connections in the tubules were distinguished. Additionally, intensive vacuolization was observed in the interstitial area of the RFR group. Cell population and connective tissue elements decreased relatively and gained a hyalinized appearance. An intense decrease was observed in the seminiferous epithelial thickness measurements of the RFR groups. Additionally, the sperm cells in the lumens of some tubules were considerably reduced under RFR.

In the literature, there are many studies related to RFR and the male reproductive system [18–19]. Most are related to the number of spermatozoa, the speed and motility of sperm cells, testis tissues, and the quality of semen. Unlike these studies, the combined effect of RFR and melatonin was investigated in the present study. With melatonin, RFR-induced reactive intermediates were evaluated by measuring the oxidant and antioxidant levels of the tissues.

Prolonged exposure of men to mobile phones changes the main sperm parameters, resulting in male infertility associated with damage to the testicular tissues. This significant consequence mentioned in the study by Agarwal et al. [18] can be clarified by three interaction mechanisms proposed for EM fields: the sensitivity of male reproductive organs to RF exposure, tissue heating associated with RF absorption, and the combined effects of both RFR sensitivity and heating [18]. Studies on the impact of RFR at different frequencies and exposure duration on male animals revealed a decrease in the count of spermatozoa [19].

In contrast to these findings, Lee et al. showed no adverse effect of long-term exposure to 845 MHz RFR on the count of sperm cells [20]. Tas and his colleagues also found no differences in testicular tissues and semen quality of male rats exposed to RFR over the long term [22].

The histological figures of this study showed similarities with findings in the literature. While there were no structural changes observed in the entire testicular tissues, the study revealed disruptions in intercellular connections and pronounced edema, which were deemed significant. It can also be concluded that the exogenous melatonin treatment reduced the adverse impacts of RFR. In our previous study, we determined increased oxidant levels and decreased antioxidant levels in brain tissues [6]. We discussed the effect of exogenous melatonin treatment on diminished oxidative stress in brain tissues. Moreover, the exogenous melatonin treatment eliminated apoptotic cell formation and structural degeneration after RFR exposure [6].

In another study, the testicular tissues of rats were exposed to Wi-Fi radiation for 1 h per day over the course of a month. While there were no significant differences in seminiferous tubule diameters and the number of pycnotic and eukaryotic cells, a decrease in Leydig cells and an increase in apoptosis were detected [22].

Several studies have shown the disruption of the basal membrane of seminiferous tubules, intense edema, and apoptotic cell formation in the spermatogonium cells under RFR exposure [23–24].

The studies mentioned an inverse ratio between RFR exposure and sperm concentration, motility, and normal morphology parameters. The boost effect of RFR on oxidative stress in semen can be evaluated by acting on plasma membrane enzymes. It is also clear that these adverse effects are related to the duration of RFR exposure and the greater risk for subjects exposed to RFR over the long term.

In the literature, many studies have shown that oxidative damage on different tissues is induced by RFR exposure. Various factors can affect this oxidative damage: exposure duration, frequency, power, polarization, and types of RFR sources, dielectric properties exposed materials, such as conductivity and resistivity of tissues under different frequencies, and the dimensions of the exposed object.

The present study investigated oxidative stress mediated by reactive intermediates and structural disruptions in testicular tissues under GSM-like RFR signals. Additionally, the study analyzed the effects of exogenous antioxidant supplements, specifically melatonin treatment, on oxidative stress induced by RFR exposure. The melatonin treatment eliminated the adverse effects of RFR exposure. Despite oxidative damage in testicular tissue and melatonin, increased antioxidant levels showed structural deterioration caused by RFR exposure.

The findings in the present study would help many researchers evaluate the interaction mechanism between RFR exposure and the male reproductive system. New studies are needed to show the possible effects of different exposure plans with different frequencies, and duration, and there is a need for quantitative data or measurements related to sperm counts, cell numbers, dilated capillary size, etc. These data will be supportive of the visual observations and interpretations.

## References

[1] Repacholi MH Low-level exposure to radiofrequency electromagnetic fields: health effects and research needs Bioelectromagnetics 1998 19 1 1 19 10.1002/(sici)1521-186x(1998)19:1<1::aid-bem1>3.3.co;2-8 9453702

[2] Yakymenko I Tsybulin O Sidorik E Henshel D Kyrylenko O Oxidative mechanisms of biological activity of low-intensity radiofrequency radiation Electromagnetic Biology and Medicine 2016 35 2 186 202 10.3109/15368378.2015.1043557 26151230

[3] Friedman J Kraus S Hauptman Y Schiff Y Seger R Mechanism of short-term ERK activation by electromagnetic fields at mobile phone frequencies Biochemical Journal 2007 405 3 559 68 10.1042/bj20061653 17456048 PMC2267306

[4] Agarwal A Desai NR Makker K Varghese A Mouradi R Effects of radiofrequency electromagnetic waves (RF-EMW) from cellular phones on human ejaculated semen: an in vitro pilot study Fertility and Sterility 2009 92 4 1318 1325 10.1016/j.fertnstert.2008.08.022 18804757

[5] Bilgici B Gün S Avcı B Akar A Engiz BK What is adverse effect of a wireless local area network, using 2.45 GHz, on the reproductive system? International Journal of Radiation Biology 2018 94 11 1054 1061 10.1080/09553002.2018.1503430 30028652

[6] Delen K Sırav B Oruç S Seymen CM Kuzay D Effects of 2600 MHz radiofrequency radiation in brain tissue of male Wistar rats and neuroprotective effects of melatonin Bioelectromagnetics 2021 42 2 159 172 10.1002/bem.22318 33440456

[7] Dong G Zhou H Gao Y Zhao X Liu Q Effects of 1.5-GHz high-power microwave exposure on the reproductive systems of male mice Electromagnetic Biology and Medicine 2021 40 2 311 320 10.1080/15368378.2021.1891091 33688776

[8] Gautam R Priyadarshini E Nirala JP Meena R Rajamani P Modulatory effects of Punica granatum L juice against 2115 MHz (3G) radiation-induced reproductive toxicity in male Wistar rat Environmental Science and Pollution Research 2021 28 39 54756 54765 10.1007/s11356-021-14378-4 34018100

[9] Gautam R Singh KV Nirala J Murmu NN Meena R Oxidative stress-mediated alterations on sperm parameters in male Wistar rats exposed to 3G mobile phone radiation Andrologia 2019 51 3 e13201 10.1111/and.13201 30461041

[10] Houston BJ Nixon B King BV De Iuliis GN Aitken RJ The effects of radiofrequency electromagnetic radiation on sperm function Reproduction 2016 152 6 R263 R276 10.1530/REP-16-0126 27601711

[11] Aitken RJ Reactive oxygen species as mediators of sperm capacitation and pathological damage Molecular Reproduction and Development 2017 84 10 1039 1052 10.1002/mrd.22871 28749007

[12] Meena R Kumari K Kumar J Rajamani P Verma HN Therapeutic approaches of melatonin in microwave radiations-induced oxidative stress-mediated toxicity on male fertility pattern of Wistar rats Electromagnetic Biology and Medicine 2014 33 2 81 91 10.3109/15368378.2013.781035 23676079

[13] Pandey N Giri S Melatonin attenuates radiofrequency radiation (900 MHz)-induced oxidative stress, DNA damage and cell cycle arrest in germ cells of male Swiss albino mice Toxicology and Industrial Health 2018 34 5 315 327 10.1177/0748233718758092 29562845

[14] Reiter RJ Tan DX Galano A Melatonin: exceeding expectations Physiology (Bethesda) 2014 29 5 325 333 10.1152/physiol.00011.2014 25180262

[15] Shokri M Shamsaei ME Malekshah AK Amiri FT The protective effect of melatonin on radiofrequency electromagnetic fields of mobile phone-induced testicular damage in an experimental mouse model Andrologia 2020 52 11 e13834 10.1111/and.13834 33040351

[16] Wood AW Loughran SP Stough C Does evening exposure to mobile phone radiation affect subsequent melatonin production? International Journal of Radiation Biology 2006 82 2 69 76 10.1080/09553000600599775 16546905

[17] Cebrián-Pérez JA Casao A Gonzàlez-Arto M dos Santos Hamilton TR Pérez- Pé R Melatonin in sperm biology: breaking paradigms Reproduction in Domestic Animals 2014 49 Suppl 4 11 21 10.1111/rda.12378 25277428

[18] Agarwal A Deepinder F Sharma RK Ranga G Li J Effect of cell phone usage on semen analysis in men attending infertility clinic: an observational study Fertility and Sterility 2008 89 1 124 128 10.1016/j.fertnstert.2007.01.166 17482179

[19] Bin-Meferij MM El-Kott AF The radioprotective effects of *Moringa oleifera* against mobile phone electromagnetic radiation-induced infertility in rats International Journal of Biology, Pharmacy and Allied Sciences 2015 8 8 12487 97 PMC4612844 26550159

[20] Lee HJ Pack JK Kim TH Kim N Choi SY The lack of histological changes of CDMA cellular phone-based radio frequency on rat testis Bioelectromagnetics 2010 31 7 528 534 10.1002/bem.20589 20607737

[21] Tas M Dasdag S Akdag MZ Cirit U Yegin K Long-term effects of 900 MHz radiofrequency radiation emitted from mobile phones on testicular tissue and epididymal semen quality Electromagnetic Biology & Medicine 2014 33 3 216 222 10.3109/15368378.2013.801850 23781998

[22] Saygin M Caliskan S Karahan N Koyu A Gumral N Testicular apoptosis and histopathological changes induced by a 2.45 GHz electromagnetic field Toxicology and Industrial Health 2011 27 5 455 463 10.1177/0748233710389851 21310776

[23] Chauhan P Verma HN Sisodia R Kesari KK Microwave radiation (2.45 GHz)-induced oxidative stress: whole-body exposure effect on histopathology of Wistar rats Electromagnetic Biology and Medicine 2016 1 11 10.3109/15368378.2016.1144063 27362544

[24] Odacı E Özyılmaz C Exposure to a 900 MHz electromagnetic field for 1 hour a day over 30 days does change the histopathology and biochemistry of the rat testis International Journal of Radiation Biology 2015 91 7 547 554 10.3109/09553002.2015.1031850 25786704

